# Scientists' warning on extreme wildfire risks to water supply

**DOI:** 10.1002/hyp.14086

**Published:** 2021-05-16

**Authors:** François‐Nicolas Robinne, Dennis W. Hallema, Kevin D. Bladon, Mike D. Flannigan, Gabrielle Boisramé, Christian M. Bréthaut, Stefan H. Doerr, Giuliano Di Baldassarre, Louise A. Gallagher, Amanda K. Hohner, Stuart J. Khan, Alicia M. Kinoshita, Rua Mordecai, João Pedro Nunes, Petter Nyman, Cristina Santín, Gary Sheridan, Cathelijne R. Stoof, Matthew P. Thompson, James M. Waddington, Yu Wei

**Affiliations:** ^1^ Canadian Partnership for Wildland Fire Science University of Alberta Edmonton Alberta Canada; ^2^ Department of Forestry and Environmental Resources North Carolina State University Raleigh North Carolina USA; ^3^ Department of Forest Engineering, Resources and Management Oregon State University Corvallis Oregon USA; ^4^ Division of Hydrologic Sciences Desert Research Institute Las Vegas Nevada USA; ^5^ Institute for Environmental Sciences University of Geneva Geneva Switzerland; ^6^ Geography and Biosciences Departments, College of Science Swansea University Swansea UK; ^7^ Department of Earth Sciences Uppsala University Uppsala Sweden; ^8^ UNEP/GRID‐Geneva Geneva Switzerland; ^9^ Department of Civil and Environmental Engineering Washington State University Pullman Washington USA; ^10^ School of Civil & Environmental Engineering University of New South Wales Kensington New South Wales Australia; ^11^ Department of Civil, Construction, & Environmental Engineering San Diego State University San Diego California USA; ^12^ South Atlantic Landscape Conservation Cooperative United States Fish and Wildlife Service Raleigh North Carolina USA; ^13^ CE3C – Centre for Ecology, Evolution and Environmental Changes, Faculdade de Ciências Universidade de Lisboa Lisbon Portugal; ^14^ Alluvium Consulting Australia Cremorne Victoria Australia; ^15^ School of Ecosystem and Forest Sciences University of Melbourne Parkville Victoria Australia; ^16^ Department of Environmental Sciences Wageningen University Wageningen the Netherlands; ^17^ Rocky Mountain Research Station, Human Dimensions USDA Forest Service Fort Collins Colorado USA; ^18^ School of Earth, Environment & Society McMaster University Hamilton Ontario Canada; ^19^ Department of Forest and Rangeland Stewardship Colorado State University Fort Collins Colorado USA

**Keywords:** climate change, extreme events, fire regime restoration, forest ecosystem services, risk governance, socio‐hydrology, water security, watershed protection

## Abstract

2020 is the year of wildfire records. California experienced its three largest fires early in its fire season. The Pantanal, the largest wetland on the planet, burned over 20% of its surface. More than 18 million hectares of forest and bushland burned during the 2019–2020 fire season in Australia, killing 33 people, destroying nearly 2500 homes, and endangering many endemic species. The direct cost of damages is being counted in dozens of billion dollars, but the indirect costs on water‐related ecosystem services and benefits could be equally expensive, with impacts lasting for decades. In Australia, the extreme precipitation (“200 mm day −1 in several location”) that interrupted the catastrophic wildfire season triggered a series of watershed effects from headwaters to areas downstream. The increased runoff and erosion from burned areas disrupted water supplies in several locations. These post‐fire watershed hazards via source water contamination, flash floods, and mudslides can represent substantial, systemic long‐term risks to drinking water production, aquatic life, and socio‐economic activity. Scenarios similar to the recent event in Australia are now predicted to unfold in the Western USA. This is a new reality that societies will have to live with as uncharted fire activity, water crises, and widespread human footprint collide all‐around of the world. Therefore, we advocate for a more proactive approach to wildfire‐watershed risk governance in an effort to advance and protect water security. We also argue that there is no easy solution to reducing this risk and that investments in both green (i.e., natural) and grey (i.e., built) infrastructure will be necessary. Further, we propose strategies to combine modern data analytics with existing tools for use by water and land managers worldwide to leverage several decades worth of data and knowledge on post‐fire hydrology.

## Graphical abstract

Wildfires are a source of socio‐hydrological extremes that increasingly threaten water security across the world; this situation may be referred to as wildfire‐watershed risks (WWR). WWR geographically vary and require locally‐adapted governance for efficient disaster risk reduction. A combination of watershed protection, forest restoration (including fire reintroduction), and adaptation of water infrastructures is the answer to reduce water supply vulnerability to wildfires.

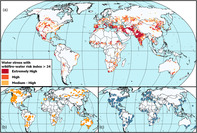

## 1 WILDFIRE RISKS TO WATER SECURITY

The 2019–2020 wildfire season in Australia was unprecedented in recorded history (Boer et al., 2020), burning several catchments supplying drinking water to the 5.5 million Sydney's inhabitants (Box 1) and threatening many aquatic species with extinction (Pittock, 2020). Similarly, the 2020 wildfires in the Western US, the greatest recorded annual area burned in the country (4.2 million hectares^1^), caused dozens of millions in damages to water distribution systems (Walton, 2020) and triggered widespread debris‐flow warnings.^2^ In both countries, heavy rainfall eventually stopped fire spread, but triggered extensive runoff, erosion, and mass movements degrading source water quality and availability, potentially for decades to come (Hanscombe, 2020; Hohner et al., 2019; Niemeyer et al., 2020; Robinne, 2020).

**FIGURE 1:**
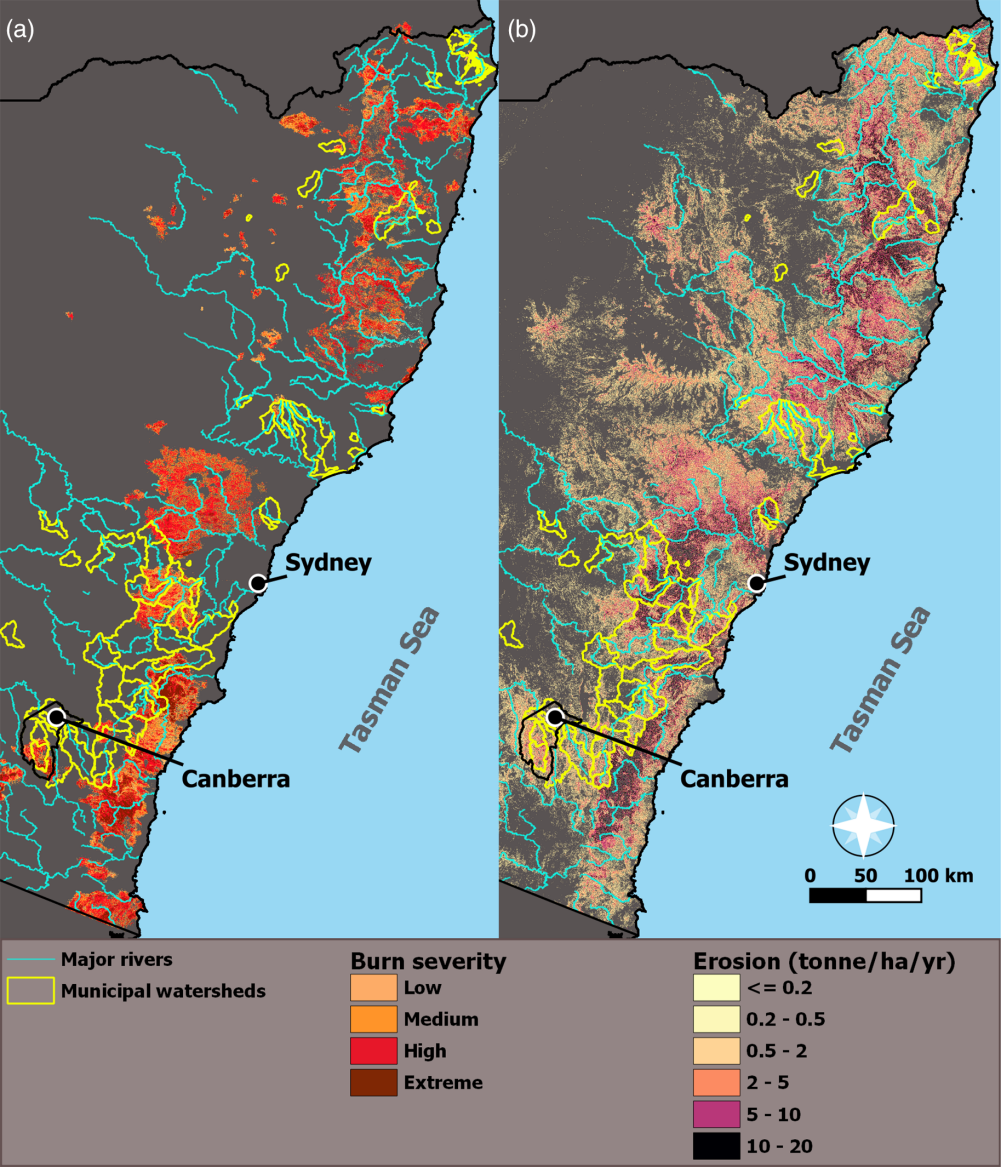
Estimated burn severity and estimated erosion in New South Wales and Australia Capital Territory. Panel (a): Wildfire severity values derived from very‐high resolution Sentinel‐2 satellite imagery using the fire extent and severity mapping algorithm from the Department of Planning, industry and environment (see Data S1). Panel (b): Estimated hillslope erosion values for the month of February 2020 calculated using the revised universal soil loss equation (RUSLE) model (see Data S1). Estimated erosion rates were higher for high burn severity. Municipal watersheds supply drinking water to Sydney and Canberra, among other communities. Data from: https://www.environment.nsw.gov.au/, https://www.seed.nsw.gov.au/, and https://data.nsw.gov.au/data/dataset

Box 1 **2019–2020 fire extent, severity, and post‐fire erosion in municipal watersheds of New South Wales and Australian Capital Territory (Figure 1).**

New South Wales was the epicentre of the 2019–2020 extreme wildfire season, experiencing 445 fires, burning ~5.8‐million hectares. The yearly average from 1989 to 2019 was 300 fires burning ~180 000 hectares, with a maximum area burned of 1.7 million hectares (computed from NSW historical fire database, see Data S1). Compared to the long‐term average, in 2019–2020 there were ~1.5‐times more fires, which burned 32‐times greater area. Most concerning, the wildfires affected source watersheds that supply drinking water for 5.5‐million people, including 25 catchments supplying Sydney and six catchments supplying Australia's capital city Canberra. In total, 46 of 78 (59%) water supply catchments burned to varying extents (14 over 50%) (see Data S1). Several townships had to restrict water consumption through boil water advisories and no‐consumption advisories. These restrictions were due, in part, to direct damage to water treatment and distribution infrastructures and to power grid damage leading to shutdown of water treatment facilities. As a result, the Australian Government pledged to commit $88.1‐million AUD to create a national disaster research centre (Australian Government, Department of Industry, Science, Energy, and Resource, 2020).

Australia and Western US extreme wildfire events, among many others, were directly linked to persistent drought and record‐breaking temperatures (Harris & Lucas, 2019; van Oldenborgh et al., 2020). As the climate becomes increasingly hotter and drier and human activities continue to expand, threats to water security will become more prevalent (AghaKouchak et al., 2018; Hallema et al., 2018; Robinne et al., 2018).

The growing overlap of extreme hydroclimatic events and expanding human activities makes water crises more likely (Franco, 2020). Climate warming is leading to greater fire danger, including in regions where fire was previously uncommon (Flannigan et al., 2013; Higuera & Abatzoglou, 2021; Shukla et al., 2019). Indeed, warmer temperatures often lead to drier fuels in which fires can ignite sooner, spread further, and burn more intensely (Flannigan et al., 2016). In rainforests and wetlands, where wet conditions usually slow or stop the spread of fires, unusually dry conditions will lead to destructive fires impacting surface waters with sediment, carbon compounds, and toxic metals (Abraham et al., 2017; Granath et al., 2016). Hence, wildfire‐watershed risks (WWR) represent a global challenge that must be addressed through proactive forest and water governance, starting with identification of areas at risk. We must then strategically apply innovative risk reduction strategies to address long‐term, large‐scale impacts from catastrophic wildfires in source watersheds (Abadi et al., 2016; Kinoshita et al., 2016).

Building on the second World Scientists' Warning to Humanity (Ripple et al., 2017), we argue that scientists, NGOs, water providers, watershed managers, fire managers, policy‐makers, and citizens share the responsibility to collect, share, and use knowledge of WWR to develop sustainable environmental policies. In this commentary, we explain the systemic nature of these risks, illustrating the need for regionally adaptive and proactive WWR governance. We also briefly review existing alternatives to WWR management, and we provide examples of forward‐thinking governance schemes in at‐risk locations.

## 2 CATASTROPHIC WILDFIRES AS SOCIO‐HYDROLOGICAL EXTREMES

Anthropogenic development has long affected the occurrence and magnitude of wildfires, droughts, and floods. These are not “true” natural hazards anymore: in many regions, most wildfires are human‐caused, often fed by excessive fuel availability resulting from past fire exclusion efforts (Pereira et al., 2019). Intensive human water use has concurrently increased the magnitude of droughts in water‐scarce regions, such as California (AghaKouchak et al., 2015), and many megacities struggle to meet growing water demand (McDonald et al., 2014). Comparatively, communities with budgetary constraints, endemic poverty, gender issues, and systemic racism are likely to be disproportionately impacted by water supply impairment (Cross, 2001; Davies et al., 2018). Hence, urban water supply is increasingly vulnerable to disruption caused by wildfires (Balch et al., 2020; Keys et al., 2019) (Box 2).

WWR is inherently systemic (Deere et al., 2017): fire is a source of socio‐hydrological extreme, whereby the dynamic interactions and dependencies between upstream source water and downstream water demand can be disrupted due to (a) exceptional wildfire magnitude, (b) vulnerability of water supply infrastructure, and (c) lack of risk governance (Di Baldassarre et al., 2018). Seeing fire as a socio‐hydrological extreme allows spatial–temporal modelling of negative wildfire impacts on water resources and the influence of WWR reduction efforts within the larger challenges of watershed management. Water resource managers are able to test various disaster risk scenarios and adjust to envisioned consequences of future wildfires—*ex ante*—and to the actual consequences of past wildfires—*ex post* (Linton & Budds, 2014). Therefore, water security and forest management are intrinsically linked; differing coping capacities of socio‐hydrosystems around the world will, however, lead to different WWR governance regimes (Di Baldassarre et al., 2013; Fischer et al., 2016; Kumar, 2015; Srinivasan et al., 2012).

### 2.1 Wildfire‐watershed risk governance

Stakeholders hold different degrees of risk knowledge, perception, and tolerance to risk (Klinke & Renn, 2012); WWR governance can account for these varying risk cultures. Effective WWR governance must also account for the compound effects of catastrophic wildfires, water supply vulnerabilities, and ecological, social, and economic stresses that can cascade towards socio‐hydrosystem collapse (Figure 3) (Balch et al., 2020; United Nations Office for Disaster Risk Reduction, 2019; van Asselt & Renn, 2011).

**FIGURE 2:**
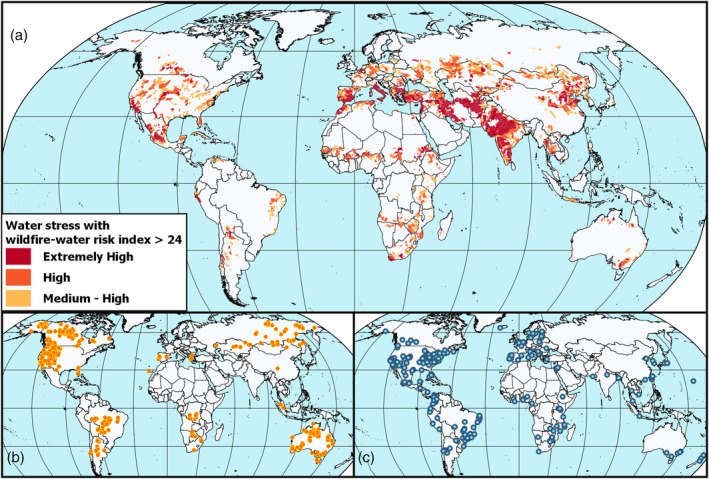
Existing and emerging global wildfire‐watershed risk hotspots. (a) Water‐stressed watersheds (i.e., annual water withdrawal exceeds annual water supply, see Data S1) with a median wildfire‐watershed risk index >24 (*n* = 8280) (Robinne et al., 2018). (b) Occurrence of extreme wildfire events recorded between 2002 to 2013 (*n* = 478; Bowman et al., 2017). (c) Cities (*n* = 252) that declared current and expected water supply challenges linked to decreasing water quantity, decreasing water quality, and/or increasing water demand (see Data S1)

**FIGURE 3:**
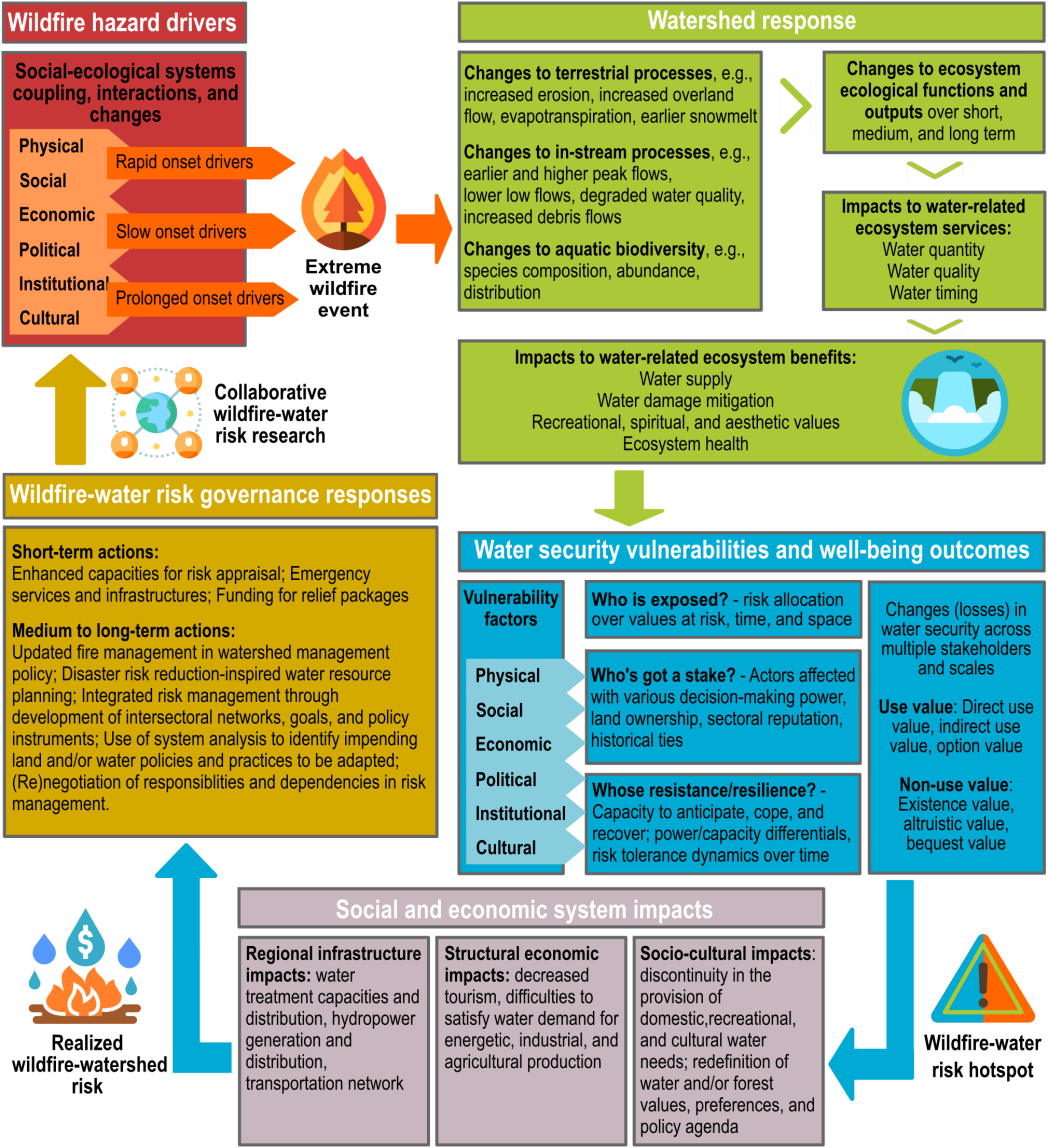
Risk governance in the wildfire‐watershed value chain. Wildfire‐watershed risks are recognized through the identification of interactions between upstream wildfire hazard (i.e., likelihood of a wildfire event of a given, potentially harmful, magnitude), watershed vulnerability, and downstream water security. After identification of water security vulnerabilities and their social and economic consequences, effective wildfire‐watershed risk governance will offer a set of options to deal with existing at‐risk situations. Rapid, slow, and prolonged onset drivers refer to the speed and depth at which changes in fire and forest management can occur: Rapid onset drivers can be acted upon quickly and have immediate effects (e.g., biomass reduction), while slow onset drivers are deeply ingrained and affect fire activity on the long term, even after changes have been made (e.g., fire exclusion policies). Icons made by Freepik and Eucalip

Community and water assets that are vulnerable to wildfire often display different risk profiles due, for instance, to different wildfire regimes. As such, post‐fire outcomes for the US and Australia do not directly apply to Chile, India, or Canada (Nunes et al., 2018). Socio‐economic development, land‐use history, public health, and relationships to water also often diverge (Linton & Budds, 2014; Miller et al., 2017; Murphy et al., 2020). Given the social‐ecological complexity involved, advancing WWR governance requires synthesis of knowledge among hydrologists, wildfire scientists, citizens, water providers, land managers, and various levels of governments responsible for watershed planning (Clark et al., 2016; Ostrom, 1996; Wheater & Gober, 2015; Parkes et al., 2010).

Understanding and embedding contextual factors is one major challenge in the development of locally‐relevant decision‐support tools for WWR governance (Blair & Buytaert, 2016; Hallema et al., 2019; Paté‐Cornell, 2012; Ruckelshaus et al., 2015). Parameterizing and adapting such tools rely heavily on data availability (Fischer et al., 2016; Kumar, 2015). Reliable hydrological data from fire‐impacted areas is hard to obtain even in economically developed countries, due to the difficulties inherent to predicting future fire activity and limited available research funds to deploy and maintain hydrological monitoring networks. Where data exist, there may be restrictive access policies and data inconsistencies. Risk governance, based on a collaborative approach to knowledge production, will help gather missing information towards the reduction of socio‐hydrosystems' vulnerability, leading to more relevant and accurate WWR‐reduction tools as a result (Canning et al., 2020; Hallema et al., 2018; Lowndes et al., 2017; Thompson et al., 2019; Wheater & Gober, 2015).

Even so, it may never be possible to exactly predict WWR (Gannon et al., 2019; O. D. Jones et al., 2014). Therefore, investing in risk prevention and literacy, as well as stakeholder preparedness through transparent communication, will help devise locally appropriate responses towards risk reduction (Boisramé et al., 2019; Kinoshita et al., 2016; McWethy et al., 2019). Working this way will help reach consensus towards the definition of regional risk profiles warranting tailored watershed policies for successful risk management; such initiatives will also facilitate the social acceptability of risk and of the actions aiming at its reduction (Blair & Buytaert, 2016; Hamilton et al., 2019; Wheater & Gober, 2015).

Box 2 **Global wildfire‐watershed risk hotspots**

Wildfire‐watershed risk hotspots are locations where water supplies and communities are susceptible to wildfire effects, such as waterworks damages (e.g., water supply infrastructure and reservoirs), loss of ecosystem services (e.g., hydropower outage, fisheries collapse), degradation of aquatic biodiversity, and potential loss of life (Dilley et al., 2005; United Nations Office for Disaster Risk Reduction, 2009) (Figure 2).

Examples of global wildfire‐watershed risk hotspots (with extreme wildfire years) include regions with:

***Humid to sub‐humid continental climate (*i.e., *temperate and boreal forests)*:** Fennoscandia (2015 wildfires), Western Canada (2016–2019), Siberia (2019). The 2016 Horse River Fire in Fort McMurray, Canada, caused ~$9 M in additional water treatment expenditures (Pomeroy et al., 2019).

***Semi‐arid climate*:** Western US (2002, 2011, 2016), Iran (2020). In 2002, the Hayman Fire in Colorado impacted Denver's water supplies, resulting in $60 M in expenditures for reservoir dredging (Bladon et al., 2014). In 2020, an unusual fire season burned 50 000 ha in the Zagros mountains of Iran; these mountains are critical for downstream water supply (Kheshti, 2020).

***Temperate oceanic climate*:** Australia (2003, 2009, 2020), Chile (2017). In 2017 in Chile, drought and poor water distribution limited firefighting capacities, disrupting water supply to millions in Chile's capital Santiago.

***Mediterranean climate*:** Western US (2013, 2018), Canada (2003), South Africa (2017), Greece (2018), Portugal (2017). In California, the 2013 Rim Fire threatened the Hetch‐Hetchy reservoir supplying water to San Francisco. The same year in Viseu, Portugal, water was diverted for firefighting, disrupting water supply for 100 000 people; water supply from several water treatment plants became limited for months due to water contamination with ash. In 2018, the post‐fire Montecito, California mudslide killed 23 persons and cost hundreds of millions in damages (Kean et al., 2019).

***Warm humid (sub)tropical climates*:** Brazil (2019), Indonesia (2015, 2019), India (2016). News reports on reduced post‐fire water quality in these regions are available, but detailed documentation of fire impacts on water resources is difficult to obtain or unavailable.

## 3 BEYOND FIREFIGHTING

Appropriate forest management maintains natural water storage and increase drought resistance, while reducing the negative impacts of unwanted fires in source watersheds (Boisramé et al., 2019; van Wagtendonk, 2007). Active forest management, including mechanical thinning and prescribed burning, can be effective at reducing fuel loads and mitigating wildfire effects (Gannon et al., 2019; K. W. Jones et al., 2017; Lydersen et al., 2017). However, fuel management alone is not a panacea due to large‐scale spatial, technical, and financial constraints (K. W. Jones et al., 2017; North, Brough, et al., 2015; Price & Bradstock, 2012). The return on investment of fuel treatment also is variable (Gannon et al., 2019; K. W. Jones et al., 2017), suggesting that complementary investments in other elements of the WWR value chain are necessary (Figure 3).

Watershed restoration offers such a complementary solution. For instance, regions where fire suppression turned historically open tree‐savannas into dense closed‐canopy forests, ecosystem water use increased significantly due to increased evapotranspiration (Boisramé et al., 2019; Roche et al., 2018), leading to reduced streamflow. Safely using naturally‐occurring wildfires to restore overstocked source watersheds can increase water security by reducing the likelihood of extreme fire events while simultaneously increasing streamflow and subsurface water storage (Boisramé et al., 2019; Roche et al., 2018); this option must, however, be carefully reviewed, as water demand from post‐fire regrowth can offset gains in water availability (Brookhouse et al., 2013; Niemeyer et al., 2020).

Degradation of wetlands and their biodiversity worldwide has led to a greater occurrence of wildfires, particularly in peat landscapes (Turetsky et al., 2015; Wilkinson et al., 2018). Restoring wetlands, biodiversity, and natural fire regimes where the ecosystems have co‐evolved with fire will help maintain the water table closer to the surface, keeping water in the landscape, preventing extreme fires, regulating water flows, and enhancing water quality (Fairfax & Whittle, 2020; Granath et al., 2016; Wilkinson et al., 2018).

Upfront investments in wildfire prevention, biomass reduction, and watershed restoration are more successful and cost‐effective than firefighting and post‐fire slope stabilization (North, Stephens, et al., 2015). Simultaneously improving watershed health and water supply infrastructures will reduce vulnerability (Box 3). Likewise, payments for ecosystem services schemes, whereby downstream communities financially support the protection of forested headwaters, are gaining traction (UNECE and FAO, 2018). There is, however, no easy solution: WWR mitigation must rely on a combination of grey (e.g., infrastructure retrofitting) and green infrastructures (e.g., watershed restoration) adapted to current and projected risk levels.

Box 3 **Building on existing WWR governance**

The following locations where WWR has been identified are proactively dealing with wildfires and their consequences.

***Melbourne, Victoria, Australia*:** Melbourne relies on forested catchments for 80% of its water supply, in which Eucalyptus forests are highly flammable. The terrain is steep and prone to high hillslope erosion rates post‐fire, leading to sediment loads >100 times greater than normal. Water entering treatment facilities is unfiltered, therefore small changes in colour and turbidity significantly impact disinfection efficacy and drinking water quality. Modelling indicates a large wildfire in the Upper Yarra Reservoir could result in water being untreatable for a year or more. Although desalinated water is available, it is expensive and cannot meet demand should the major water supply catchment go offline due to fire‐caused contamination. Thus, Melbourne Water has invested millions in research programs to inform fuel reduction, firefighting efforts, and post‐fire response (Canning et al., 2020).

***Sydney, New South Wales, Australia*:** The extensive 2019–2020 wildfires burned 35% of Sydney's largest water supply catchment, Warragamba. In 2019, the Greenwattle Creek fire was followed by intense rainfall exceeding 200 mm in one day, resulting in substantial ash and sediment transfer into the water storage, Lake Burragorang. Hence, Sydney Water led risk mitigation efforts—ash, sediment, and contaminant transfer modelling into the lake; sediment plume monitoring; water quality monitoring. The vertically flexible design of the offtake at the dam wall enabled water to be extracted from outside of the plume zone (Canning et al., 2020).

***Denver, Colorado, USA*:** The Upper South Platte watershed (~6900 km^2^) provides 80% of the municipal water supply. Denver Water delivers water to 1.4‐million customers. After wildfires impacted water supply and distribution in the 1990s, multi‐stakeholder partnerships were started to plan and implement watershed‐scale wildfire risk assessments, fuel reduction, and ecosystem restoration. The Upper South Platte provides an example of watershed investment programs, or payment for ecosystem services focused on wildfire risk reduction for the protection of water supplies. Through the US Forest Service Forests to Faucets program, Denver Water invested $16.5‐million, partly covered by a $27 fee charged to each household served by the utility. In the first five years of the program, wildfire mitigation measures were applied to over 470 000 hectares in the watershed.

***Fort Collins, Colorado, USA*:** Following the 2012 High Park fire in the source watershed of Fort Collins, the municipality closed the river water intake and relied on a secondary reservoir. The installation of an in‐stream turbidity sensor expanded upstream monitoring and provided an early warning system for water treatment operators; unusual turbidity signals triggered intake pipeline shut down, protecting conveyance infrastructure from destructive debris flows and sediment overloads. Additionally, the municipality constructed a pre‐sedimentation basin to dampen the effects of post‐fire turbidity loads on treatment process performance. Collectively, these measures allowed for continuous delivery of safe drinking water. Fifteen partner organizations are now working together towards forest restoration in upstream watersheds through the Northern Front Range Collaborative Watershed Resilience Project. In 2020, the municipal water supply was again threatened by the Cameron Peak Fire, which burned ~85 000 hectares in the Poudre River watershed and triggered water restrictions.^3^

***Manchester, United Kingdom*:** The 2018 Saddleworth Moor wildfire was one of England's largest in recorded history, burning over 18 km^2^ of upland terrain that supplies the greater Manchester area with drinking water and has elevated heavy metal concentrations from past industrial activity. The water supplier, United Utilities, acted promptly in collaboration with scientists, treating burned hillslopes and gullies with biodegradable erosion prevention measures. This also prompted modelling contamination potential from future fires in unburned catchments in this region for future risk mitigation.

***Lisbon, Portugal*:** Fires in 2013 and 2017 threatened water supplies of Lisbon, Portugal, managed by Empresa Portuguesa das Águas Livres. After 2013, an additional water treatment line was built to address potential short‐term water quality issues. After 2017, key parts of the watersheds received emergency slope stabilization. Ongoing monitoring and numerical modelling efforts will help better assess WWR and evaluate the cost‐effectiveness of pre‐fire watershed management, post‐fire slope intervention, and water treatment measures.

***Western Cape Province, Republic of South Africa*:** The “Working for Water programme” focuses on invasive alien vegetation clearing for water provision. It directly links to wildfire risk management as many invasive species (e.g., the tree species *Acacia mearnsii*) increase fuel loads and connectivity, allowing fire to spread into riparian zones at higher rates and severity. This poses a threat for water quality and freshwater ecosystem health as it degrades bank stability and increases erosion, leading to excess sediment and ash delivery into streams. This case exemplifies the enhancement of positive feedbacks between ecological perturbations (invasive species and wildfire) in the current context of global change. A standard practice within this program is the creation of firebreaks to reduce wildfire risk.

## 4 CONCLUSION

Planning for a future where watershed response to wildfire are highly non‐stationary will be challenging (O'Connor et al., 2017). Stakeholders involved in water security must actively seek a better socio‐hydrological understanding of existing wildfire hazards in their water‐supply areas. Research and management efforts are particularly urgent in areas where WWR are emerging and where wildfire and hydrological sciences remain underfunded. To this end, advancing WWR governance will promote collaboration and knowledge transfer, prevention, and preparedness to face extreme wildfire events and cascading disastrous consequences on water ecosystem services, like those experienced in Australia, Western North America, and elsewhere (Khan et al., 2015; Martin, 2016; McWethy et al., 2019; Robinne et al., 2018).

## Supporting information


**Data S1.** Supporting Information

## Data Availability

Datasets from Robinne et al. 2018 and Bowman et al. 2017 used to create figure 2 are available upon direct request to the authors. All other datasets used in this paper are open‐access and available online; Internet links to those datasets are provided in figure captions and in the supplementary material describing data processing. Note that these open‐access datasets are not managed by any of the co‐authors and that access policies set by the publishers may change.

## ENDNOTES

^1^
https://www.nifc.gov/fireInfo/fireInfo_stats_totalFires.html.

^2^
https://landslides.usgs.gov/hazards/postfire_debrisflow/.

^3^
https://www.fcgov.com/utilities/water-restrictions/.
