# Supplementary material for: Scientists' warning on extreme wildfire risks to water supply
Source: Hydrol Process. 2021 May 16;35(5):e14086. doi: 10.1002/hyp.14086 (PMC8251805; doi:10.1002/hyp.14086)
Supplement: Supplementary file 1 — Data S1. Supporting Information [file HYP-35-0-s001.zip › 20200803_RobinneEtAl_WildfireWaterSecurity_Supplementary/Date_RobinneEtAl_WildfireWaterSecurity_Supplementary.pdf]

# Bushfire disaster sends dire warning to global water community - Supplementary material

## Figure 1: Estimated burn severity and estimated erosion in New South Wales and Australia Capital Territory.

Data for Figure 1 came from various open-data sources. Only information essential to further understand Figure 1 are provided. Detailed information (i.e., metadata) on the material listed below is available by following the links provided in the References section of this document.

The Australian Government provided:

- A surface hydrology vector layer used to display the main rivers [1]. Only major perennial river segments were selected for display;
- An State boundaries vector layer used to display administrative boundaries for NSW and ACT [2].

The Government of New South Wales provided:

- A vector layer of drinking water catchments (i.e., municipal watersheds) [3];
- A raster layer of fire extent and severity derived from aerial and satellite imagery [4]. Fire severity is defined as “a metric of the loss of biomass caused by fire, both wildfires and hazard reduction burns”. Low fire refers to “burned understory with unburned canopy”; moderate fire severity refers to “partial canopy scorch”; high fire severity refers to “complete canopy scorch”; and extreme refers to “complete canopy consumption” [5];
- A raster layer of modelled hillslope erosion for February 2020 taking into account change in lad cover due to the fires [6]. Erosion levels are provided as estimated soil loss in tonne $ha^{-1}$ $year^{-1}$ calculated using the Revised Universal Soil Loss Equation (i.e., RUSLE) [7].

The Australian Capital Territory Government provided:

- A vector layer of Water Management Areas (i.e., municipal watersheds) [8].

The location of Canberra and Sydney was extracted from a free vector of populated places at the 1:10m-scale provided by Natural Earth (https://www.naturalearthdata.com/downloads/10m-cultural-vectors/).

Data were assembled into a map using QGIS 3.10.0 [9] and displayed in the Geocentric Datum of Australia 1994 coordinate system (EPSG:4283). No new data was created for this figure besides the one for municipal watersheds (see next section on fire statistics).

## Box 1: Fire statistics and area burned in municipal watersheds

Basic wildfire statistics were computed using the layer of Fire history of NSW [10]. We calculated the area burned and the number of fires for the 2019-2020 fire season and compared it with the 1989-2018 long-term average. Statistics were computed using R3.6.3 language for statistical computing [11] within the RStudio Integrated Development Environment [12] (See the script “NSW-ACT_AreaBurned_Per_Catchment.R” in the Supplementary Code/BOX_1 folder). Readers/users must

keep in mind that the fire polygon dataset was of rather low quality; for instance, it is common that a single fire was divided in multiple small polygons whose geometry was hard to reconcile; in many cases, fires did not have a unique identifier and fire dates were absent or illogical (fire end-date earlier than start-date). The only consistent information was the fire season year (e.g., 2019-2020). As errors were spread across the whole dataset, it was used as is; this is why we recommend that the fire statistics that were thereby collected and reported are interpreted with caution.

We then computed the area burned per municipal watershed for the 2019-2020 wildfire season. To do so, a layer of municipal watersheds was first created by merging source watersheds polygons for ACT and NSW [3,8]. Beforehand, NSW watershed layer needed to be manually cleaned because of serious geometric and attribute errors that hindered the calculation of area burned statistics per basin:

1) Watersheds with the same name but with apparent non-hydrological divisions (e.g., local government boundaries) were merged together. The cleaning focused on merging multiple parts into one catchment based on obvious physical catchment boundaries. Existing single parts that were distant from the main catchment were kept as individual polygons. In sum, each polygon was assessed for its size and shape in order to determine if it should be deleted, left alone, or merged with neighbouring polygon. Only multipart catchments were left as is when their size was >10 ha, although in several cases watershed boundaries were not obvious and errors might still exist.
2) For Byron watershed, all polygons <10 ha were removed ($n$ = 80). This is an estuary; the geometry is thus very complicated but the overall shape was not affected by the removal of these small polygons.
3) Several watersheds from the NSW layer overlapped with watersheds polygons from ACT. They were removed. A minimal amount of overlap remained between ACT and NSW watersheds, and the potential impact on area burned calculation (i.e., double-counting) was deemed marginal.
4) Polygon holes were removed as well, using a 0.00001 threshold.

The polygon layer for ACT was clean. Once the manual cleaning was done, NSW layer was combined with ACT layer. Area in hectares was recomputed and field catchment name was put in upper case. However, there was not enough details provided in the original layers to assign an individual name or identifier to each watershed or their parts. The clean watershed layer is provided in the supplementary data folder ("ACT_NSW_Municipal_Catchments.shp"). This layer was also used to represent municipal watersheds in Figure 1, as well as to extract 2019-2020 wildfire perimeters ("Area_Burned_Catchment_2019_2020.shp" in the Supplementary/BOX_1 folder). All geoprocessing operations (i.e., cleaning and merging of GIS layers) were done in QGIS 3.10.0 [9] and final wildfire statistics were computed using R3.6.3 language for statistical computing [11] within the RStudio Integrated Development Environment [12] (See the script "NSW-ACT_AreaBurned_Per_Catchment.R" in the Supplementary Code folder).

# Figure 2: Global wildfire-watershed risk hotspots

Panel (a) displays a subset of Aqueduct 3.0, an open-access global watershed dataset produced by the World Resource Institute (WRI) [13]. This dataset was developed using the WRI's water risk framework, a state-of-the-art approach that merges together hydrological modelling and numerous environmental datasets to produce a set of water security indicators and water risk scores available worldwide [14]. Our subset was created based on two main criteria:

1) A baseline water stress score between 2 and 5, according to 1960-2014 hydrological and water use records. Water stress is "the ratio of total water withdrawals to available renewable surface and

groundwater supplies" (see p.4 in ref. [14]). These scores represent medium to very-high water stress hazard, with higher values exacerbating competition between water uses. Limitations of this indicator are detailed in Aqueduct 3.0's documentation [14].

2) A GWWRI score of 24, which is the median score value of the Global Wildfire-Water Risk Index (GWWRI), created by Robinne et al. [16]. The GWWRI is a global composite index of wildfire risk to water security, inspired by earlier effort to map global threats to water security based on a multi-criteria approach [17]. Median value was calculated for each Aqueduct 3.0 watershed using the Zonal Statistics tool in QGIS 3.10.0. The dataset is available upon request to the authors of the original dataset.

The data associated with this panel, "Aqueduct_BaselineWaterStress.shp", is provided in the folder "Supplementary_Data\FIGURE_2\PANEL_A". We kept all the attributes necessary to identify each watershed, as well as the field "bws_label" that indicates the baseline water stress of each watershed in plain text. Further details about the structure of the attribute table can be found in the Aqueduct 3.0's metadata [15]. This vector file also contains two additional attributes, GWWRI_Mn and GWWRI_Md, which are the GWWRI mean and median per watershed, respectively.

Panel (b) shows extreme wildfire events, or EWEs, recorded by Bowman et al. [18] between 2002 and 2013. Fire events were recorded as EWE when they exceeded the 99.997$^{th}$ percentile of the daily sum of fire radiative power per 100 km$^2$, expressed in megawatts, calculated using the Moderate Resolution Imaging Spectroradiometer (MODIS) Fire Radiative Power times series [19]. The dataset is available upon request to the authors of the original dataset.

Panel (c) shows communities (e.g., cities, towns) that have reported existing and/or short-term risks to their water supply due to declining water quality and increased water stress or scarcity, in 2018. Data is a subset of a larger open dataset provided by CDP Worldwide and downloaded on March the 3$^{rd}$ of 2020 [20]. The original dataset came in a csv format with a number of minimal formatting errors (e.g., city names spelled differently, inversed longitude and latitude). The dataset was small enough (i.e., 608 rows) for us to proceed with manual correction using a spreadsheet editor. We then used location information in longitude and latitude to create a GIS layer using R3.6.3 language for statistical computing [11] within the RStudio Integrated Development Environment [12] (See the script "2018_Cities_Water_Risks_CDP.R" in the Supplementary Code/BOX_2 folder). The final dataset used for display in Figure 2 is provided in the Supplementary_Data folder ("2018_Cities_Water_Risks_Stress_Quality_Current_ShortTerm.shp").

Data were assembled into a map using QGIS 3.10.0 [9] and displayed in the World Robinson coordinate system (ESRI:54030).

# References


1. Crossman, S. & Li, O. *Surface Hydrology Lines (Regional)*. http://pid.geoscience.gov.au/dataset/ga/83107 (2015).

2. Australian Government. *State Boundaries FEBRUARY 2020*. https://data.gov.au/data/dataset/bdcf5b09-89bc-47ec-9281-6b8e9ee147aa/resource/136ff1ca-b258-4641-9592-f933367bba6e/download/state-boundaries-february-2020.zip (2020).

3. New South Wales Government. *Environmental Planning Instrument - Drinking Water Catchment*. https://www.planningportal.nsw.gov.au/opendata/dataset/65812163-854f-49c9-8402-fe30a6778353 (2016).

4. Department of Planning, Industry and Environment. *Fire Extent and Severity Mapping (FESM)*.

https://datasets.seed.nsw.gov.au/dataset/fire-extent-and-severity-mapping-fesm (2020).

5. Department of Planning, Industry and Environment. *NSW Fire and the Environment 2019-2020 Summary - Biodiversity and landscape data and analyses to understand the effects of the fire events*. (2019).

6. Department of Planning, Industry and Environment. *Modelled Hillslope Erosion over New South Wales*. https://datasets.seed.nsw.gov.au/dataset/modelled-hillslope-erosion-over-new-south-wales (2020) doi:10.25948/5f0bd19c72d76.

7. Yang, X. *et al.* Digital mapping of soil erodibility for water erosion in New South Wales, Australia. *Soil Res.* **56**, 158 (2018).

8. Australian Capital Territory Government. *ACT Water Management Areas*. http://data.actmapi.act.gov.au/arcgis/rest/services/actmapi/basic/MapServer/88 (2013).

9. QGIS.org. QGIS Geographic Information System. (2020).

10. Government of New South Wales. NPWS Fire History - Wildfires and Prescribed Burns. (2020).

11. R Core Team. R: A Language and environment for Statistical Computing. (2019).

12. RStudio Team. RStudio: Integrated Development for R. (2015).

13. World Resource Institute. *Aqueduct Global Maps 3.0 Data*. https://www.wri.org/resources/data-sets/aqueduct-global-maps-30-data (2019).

14. Hofste, R. W. *et al. Aqueduct 3.0: Updated Decision-Relevant Global Water Risk Indicators*. https://www.wri.org/publication/aqueduct-30 (2019).

15. World Resource Institute. Aqueduct 3.0 Water Risk Atlas Metadata. https://github.com/wri/aqueduct30_data_download/blob/master/metadata.md (2019).

16. Robinne, F.-N. *et al.* A spatial evaluation of global wildfire-water risks to human and natural systems. *Sci. Total Environ.* **610–611**, 1193–1206 (2018).

17. Vörösmarty, C. J. *et al.* Global threats to human water security and river biodiversity. *Nature* **467**, 555–561 (2010).

18. Bowman, D. M. J. S. *et al.* Human exposure and sensitivity to globally extreme wildfire events. *Nat. Ecol. Evol.* **1**, 0058 (2017).

19. Giglio, L., Descloitres, J., Justice, C. O. & Kaufman, Y. J. An Enhanced Contextual Fire Detection Algorithm for MODIS. *Remote Sens. Environ.* **87**, 273–282 (2003).

20. Zehra, D. *2018 - Cities Water Risks*. https://data.cdp.net/Water/2018-Cities-Water-Risks/j79t-an58 (2019).
